# Supplementary material for: Multi‐dataset identification of innovative feature genes and molecular mechanisms in keratoconus
Source: J Cell Mol Med. 2024 Sep 19;28(18):e70079. doi: 10.1111/jcmm.70079 (PMC11412914; doi:10.1111/jcmm.70079)
Supplement: Supplementary file 2 — Material S2. [file JCMM-28-e70079-s002.zip › 7.GSEA analysis/5.RNF217-AS1/KEGG_PROTEASOME.html]

Details for gene set KEGG\_PROTEASOME[GSEA]

|  || Dataset | RNF217-AS1.group.cls#h\_versus\_l |
| Phenotype | group.cls#h\_versus\_l |
| Upregulated in class | h |
| GeneSet | KEGG\_PROTEASOME |
| Enrichment Score (ES) | 0.23716693 |
| Normalized Enrichment Score (NES) | 0.85407525 |
| Nominal p-value | 0.6653226 |
| FDR q-value | 1.0 |
| FWER p-Value | 1.0 |
Table: GSEA Results Summary

  

Fig 1: Enrichment plot: KEGG\_PROTEASOME      
 Profile of the Running ES Score & Positions of GeneSet Members on the Rank Ordered List

  

| SYMBOL | TITLE | RANK IN GENE LIST | RANK METRIC SCORE | RUNNING ES | CORE ENRICHMENT || 1 | PSMB11 | na | 474 | 0.537 | 0.2372 | Yes |
| 2 | PSMA4 | na | 9655 | 0.092 | 0.0156 | No |
| 3 | PSMC2 | na | 10278 | 0.082 | 0.0358 | No |
| 4 | PSMD3 | na | 11177 | 0.068 | 0.0414 | No |
| 5 | PSMA1 | na | 11231 | 0.067 | 0.0711 | No |
| 6 | PSME4 | na | 12490 | 0.049 | 0.0576 | No |
| 7 | PSMD13 | na | 13925 | 0.032 | 0.0310 | No |
| 8 | PSMC6 | na | 14095 | 0.030 | 0.0399 | No |
| 9 | PSMB3 | na | 15186 | 0.017 | 0.0165 | No |
| 10 | PSMD12 | na | 15263 | 0.016 | 0.0220 | No |
| 11 | PSMD8 | na | 15538 | 0.014 | 0.0204 | No |
| 12 | PSME1 | na | 15835 | 0.010 | 0.0168 | No |
| 13 | PSMB7 | na | 16118 | 0.008 | 0.0122 | No |
| 14 | PSMA7 | na | 16202 | 0.007 | 0.0129 | No |
| 15 | PSMC5 | na | 16366 | 0.005 | 0.0106 | No |
| 16 | PSMD4 | na | 16430 | 0.004 | 0.0108 | No |
| 17 | PSMD2 | na | 16462 | 0.004 | 0.0118 | No |
| 18 | PSMA3 | na | 16742 | 0.001 | 0.0043 | No |
| 19 | PSMB10 | na | 16743 | 0.001 | 0.0048 | No |
| 20 | PSMD14 | na | 17171 | -0.003 | -0.0059 | No |
| 21 | PSMB4 | na | 17260 | -0.004 | -0.0065 | No |
| 22 | PSMD11 | na | 17473 | -0.006 | -0.0098 | No |
| 23 | PSMB8 | na | 18056 | -0.011 | -0.0213 | No |
| 24 | PSMB2 | na | 18135 | -0.012 | -0.0179 | No |
| 25 | PSMC3 | na | 18573 | -0.016 | -0.0230 | No |
| 26 | PSMD7 | na | 18585 | -0.016 | -0.0157 | No |
| 27 | PSMA6 | na | 18657 | -0.017 | -0.0098 | No |
| 28 | PSMA2 | na | 18913 | -0.019 | -0.0081 | No |
| 29 | PSMB5 | na | 18915 | -0.019 | 0.0009 | No |
| 30 | PSMB1 | na | 18973 | -0.020 | 0.0084 | No |
| 31 | PSME2 | na | 19178 | -0.022 | 0.0127 | No |
| 32 | PSMD6 | na | 19327 | -0.023 | 0.0194 | No |
| 33 | PSMC1 | na | 19546 | -0.026 | 0.0251 | No |
| 34 | PSMD1 | na | 19982 | -0.030 | 0.0265 | No |
| 35 | PSMA8 | na | 20103 | -0.031 | 0.0375 | No |
| 36 | PSMB6 | na | 20263 | -0.033 | 0.0481 | No |
| 37 | PSMA5 | na | 20589 | -0.036 | 0.0555 | No |
| 38 | PSMC4 | na | 21008 | -0.040 | 0.0621 | No |
| 39 | PSME3 | na | 21882 | -0.049 | 0.0600 | No |
| 40 | PSMF1 | na | 23206 | -0.064 | 0.0518 | No |
| 41 | PSMB9 | na | 23572 | -0.069 | 0.0737 | No |
| 42 | POMP | na | 27651 | -0.140 | 0.0215 | No |
| 43 | IFNG | na | 33359 | -0.391 | 0.0395 | No |
Table: GSEA details [plain text format]

  

Fig 2: KEGG\_PROTEASOME      
 Blue-Pink O' Gram in the Space of the Analyzed GeneSet

  

Fig 3: KEGG\_PROTEASOME: Random ES distribution      
 Gene set null distribution of ES for **KEGG\_PROTEASOME**
